# Punctaporonins N–S, New Caryophyllene Sesquiterpenoids from* Cytospora* sp.

**DOI:** 10.1155/2017/7871459

**Published:** 2017-10-24

**Authors:** Yan Li, Qingbin Wang, Xingzhong Liu, Yongsheng Che

**Affiliations:** ^1^State Key Laboratory of Toxicology & Medical Countermeasures, Beijing Institute of Pharmacology & Toxicology, Beijing 100850, China; ^2^Institute of Vegetables and Flowers, Chinese Academy of Agricultural Sciences, Beijing 100081, China; ^3^State Key Laboratory of Mycology, Institute of Microbiology, Chinese Academy of Sciences, Beijing 100190, China

## Abstract

Six new caryophyllene sesquiterpenoids, punctaporonins N–S (**1**–**6**), and three known ones, 6-hydroxypunctaporonins B (**7**), A (**8**), and E (**9**), have been isolated from solid cultures of* Cytospora *sp. The structures of** 1**–**6** were elucidated primarily by NMR spectroscopy. The absolute configuration of** 1 **was assigned by X-ray crystallographic analysis of its *S*-MTPA ester. Compounds** 2**,** 5**, and** 6** showed modest cytotoxicity against HeLa cells.

## 1. Introduction

**Figure 1:**
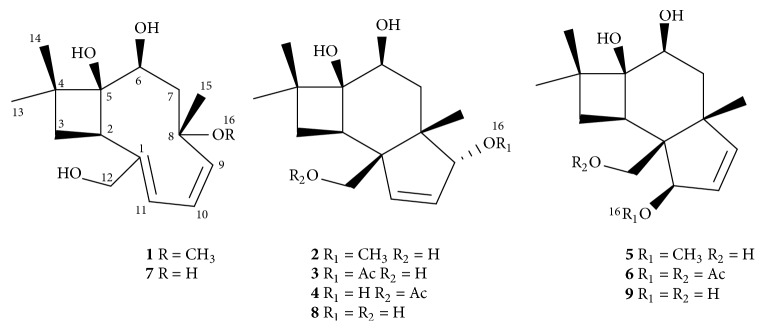
Structures of compounds** 1**–**9**.

Fungi are capable of producing a variety of bioactive secondary metabolites [1]. Since the secondary metabolism of fungi may be influenced by selection pressures exerted by other organisms and the environment in which they reside, those species thriving in unique and competitive niches are especially likely to produce bioactive natural products with diverse and interesting structural features [2, 3]. Based on this consideration and the documented success in finding new bioactive natural products from special types of fungi [4], we initiated chemical studies of the fungi inhabiting either the fruiting body and larvae of* Cordyceps sinensis* [5–10] or its surface soil [11–13]. As an extension, we also studied those species isolated from the soil samples that were collected on the Qinghai-Tibetan plateau at altitudes above 3,200 m, the environment in which* Cordyceps sinensis* was typically found. During the course of our continuing search for new bioactive natural products from this kind of fungal species, an ascomycetous fungus* Cytospora *sp. was isolated from a soil sample that was collected at Linzhi, Tibet, China. Our initial investigation of this fungus that was fermented in rice medium at 25°C led to the isolation of three antimicrobial caryophyllene-derived meroterpenoids [14, 15] and three cytotoxic caryophyllene sesquiterpenoids [16]. In the current work, the fungus was refermented in the same solid culture medium at 15°C, and the HPLC chromatogram of the crude extract revealed the presence of additional components. Fractionation of an EtOAc extract afforded six new caryophyllene sesquiterpenoids, which we named punctaporonins N–S (**1**–**6**), and three known ones, 6-hydroxypunctaporonins B (**7**), A (**8**), and E (**9**) (Figure 1) [17]. Details of the isolation, structure elucidation, and cytotoxicity of these compounds are described herein.

## 2. Materials and Methods

### 2.1. General Experimental Procedures

Optical rotations were measured on a Perkin-Elmer 241 polarimeter, and UV data were recorded on a Shimadzu Biospec-1601 spectrophotometer. IR data were recorded using a Nicolet Magna-IR 750 spectrophotometer. ^1^H and ^13^C NMR data were acquired with Varian Mercury-400, Varian Mercury-500, and Varian Mercury-600 MHz spectrometers using solvent signals (acetone-*d*_6_: *δ*_H_ 2.05/*δ*_C_ 29.8, 206.1; pyridine-*d*_5_: *δ*_H_ 7.21, 7.58, 8.73) as references. The HMQC and HMBC experiments were optimized for 145.0 and 8.0 Hz, respectively. ESIMS data and HRESIMS data were obtained using an Agilent Accurate-Mass-Q-TOF LC/MS 6520 instrument equipped with an electrospray ionization (ESI) source. The fragmentor and capillary voltages were kept at 125 and 3500 V, respectively. Nitrogen was supplied as the nebulizing and drying gas (300°C). The flow rate of the drying gas and the pressure of the nebulizer were 10 L/min and 10 psi, respectively. All MS experiments were performed in positive ion mode. Full-scan spectra were acquired over a scan range of* m/z* 100–1000 at 1.03 spectra/s. All solvents used were of analytical grade. Column chromatography was performed with silica gel (100–200 or 200–300 mesh, Qingdao Marine Chemical Inc., China) and Sephadex LH-20 (GE, USA). Semipreparative HPLC was performed on an Agilent 1260 G1311C isopump equipped with a G1365D MWD detector and an Agilent Zorbax SB-C_18_ column (5 *μ*m; 9.40 mm × 250 mm).

### 2.2. Fungal Material and Fermentation

The isolation, identification, and fermentation of the fungus* Cytospora* sp. were the same as those we previously described [14–16], except the incubation temperature was changed to 15°C.

### 2.3. Extraction and Isolation

The fermented material was extracted with EtOAc (4 × 1.0 L), and the organic solvent was evaporated to dryness under vacuum to afford a crude extract (4.2 g). The extract was fractionated by silica gel VLC using petroleum ether–EtOAc gradient elution. The fraction (100 mg) eluted with 35% EtOAc was separated by Sephadex LH-20 column chromatography (CC) eluting with 1 : 1 CH_2_Cl_2_–MeOH. The resulting subfractions were combined and further purified by semipreparative RP HPLC (59% MeOH in H_2_O for 35 min; 2 mL/min) to afford punctaporonins N (**1**; 4.5 mg, *t*_R_ 21.22 min), O (**2**; 3.5 mg, *t*_R_ 31.33 min), P (**3**; 2.0 mg, *t*_R_ 14.05 min), and R (**5**; 0.8 mg, *t*_R_ 24.22 min). The fraction (50 mg) eluted from the silica gel column with 45% EtOAc was purified by RP HPLC (35% CH_3_CN in H_2_O for 20 min; 2 mL/min) to afford punctaporonin Q (**4**; 5.0 mg; *t*_R_ 16.44 min). The fraction (35 mg) eluted from the silica gel column with 40% EtOAc was purified by RP HPLC (40% CH_3_CN in H_2_O for 20 min; 2 mL/min) to afford punctaporonin S (**6**; 2.0 mg; *t*_R_ 17.02 min). The fraction (150 mg) eluted from the silica gel column with 50% EtOAc was fractionated again by Sephadex LH-20 CC eluting with 1 : 1 CH_2_Cl_2_–MeOH. Purification of the resulting subfractions with different gradients afforded 6-hydroxypunctaporonins B (**7**; 10.0 mg, *t*_R_ 23.61 min; 50% MeOH in H_2_O for 30 min), A (**8**; 8.4 mg, *t*_R_ 16.12 min; same gradient as in purification of** 7**), and E (**9**; 3.5 mg, *t*_R_ 14.83 min; same gradient as in purification of** 7**).

### 2.4. Identification

*Punctaporonin N (* ***1***). White powder (MeOH); [*α*]^25^_D_ –73 (*c* 0.1, MeOH); UV (MeOH) *λ*_max_ (log⁡*ε*) 198 (3.40) nm; IR (neat) *ν*_max_ 3317 (br), 2936, 2870, 1736, 1714, 1463, 1372, 1241, 1095, 1037 cm^−1^; ^1^H, ^13^C NMR, HMBC, and NOESY data see Table 1; (+)–HR–ESI–MS* m/z* 305.1719 [M + Na]^+^ (calcd. for C_16_H_26_O_4_Na, 305.1723).

*Punctaporonin O (* ***2***). Colorless oil (MeOH); [*α*]^25^_D_ –47 (*c* 0.1, MeOH); UV (MeOH) *λ*_max_ (log⁡*ε*) 202 (3.58) nm; IR (neat) *ν*_max_ 3263 (br), 2932, 2875, 1713, 1463, 1361, 1181, 1089, 1058, 1029 cm^−1^; ^1^H and ^13^C NMR data see Table 2; (+)–HR–ESI–MS* m/z* 305.1721 [M + Na]^+^ (calcd. for C_16_H_26_O_4_Na, 305.1723).

*Punctaporonin P (* ***3***). Colorless oil (MeOH); [*α*]^25^_D_ –71 (*c* 0.1, MeOH); UV (MeOH) *λ*_max_ (log⁡*ε*) 203 (3.55) nm; IR (neat) *ν*_max_ 3262 (br), 2958, 2935, 2872, 1735, 1717, 1372, 1240, 1089, 1021 cm^−1^; ^1^H and ^13^C NMR data see Table 2; (+)–HR–ESI–MS* m/z* 333.1669 [M + Na]^+^ (calcd. for C_17_H_26_O_5_Na, 333.1672).

*Punctaporonin Q (* ***4***). Colorless oil (MeOH); [*α*]^25^_D_ –37 (*c* 0.1, MeOH); UV (MeOH) *λ*_max_ (log⁡*ε*) 203 (3.60) nm; IR (neat) *ν*_max_ 3438 (br), 2957, 2936, 1730, 1715, 1381, 1249, 1031 cm^−1^; ^1^H and ^13^C NMR data see Table 2; (+)–HR–ESI–MS* m/z* 333.1676 [M + Na]^+^ (calcd. for C_17_H_26_O_5_Na, 333.1672).

*Punctaporonin R (* ***5***). Colorless oil (MeOH); [*α*]^25^_D_ –30 (*c* 0.1, MeOH); UV (MeOH) *λ*_max_ (log⁡*ε*) 200 (3.22) nm; IR (neat) *ν*_max_ 3256 (br), 2957, 2934, 2869, 1711, 1462, 1365, 1102, 1057, 1031, 984 cm^−1^; ^1^H and ^13^C NMR data see Table 3; (+)–HR–ESI–MS* m/z* 305.1717 [M + Na]^+^ (calcd. for C_16_H_26_O_4_Na, 305.1723).

*Punctaporonin S (* ***6***). Colorless oil (MeOH); [*α*]^25^_D_ –84 (*c* 0.1, MeOH); UV (MeOH) *λ*_max_ (log⁡*ε*) 202 (3.56) nm; IR (neat) *ν*_max_ 3486 (br), 2953, 2938, 2871, 1736, 1374, 1252, 1040, 990 cm^−1^; ^1^H and ^13^C NMR data see Table 3; (+)–HR–ESI–MS* m/z* 375.1781 [M + Na]^+^ (calcd. for C_19_H_28_O_6_Na, 375.1778).

*6-Hydroxypunctaporonin B (* ***7***). ^1^H, ^13^C NMR, and the MS data were consistent with literature values [17].

*6-Hydroxypunctaporonin A (* ***8***). ^1^H, ^13^C NMR, and the MS data were consistent with literature values [17].

*6-Hydroxypunctaporonin E (* ***9***). ^1^H, ^13^C NMR, and the MS data were consistent with literature values [17].

### 2.5. Preparation of (*S*)-MTPA Ester (**1a**)

A sample of** 1** (2.0 mg, 0.007 mmol) was dissolved in pyridine (2.0 mL) in a 10 mL round-bottomed flask. (*R*)-MTPA Cl (2.0 *μ*L, 0.011 mmol) were quickly added, and the flask was sealed and all contents were stirred at room temperature for 12 h. The mixture was evaporated to dryness and purified by semipreparative HPLC (90% CH_3_OH in H_2_O for 20 min; 2 mL/min) to afford** 1a** (2.2 mg, *t*_R_ 15.72 min): colorless platelets; ^1^H NMR (pyridine-*d*_5_, 500 MHz)*δ* 6.16 (1H, s, H-11), 6.00 (1H, d,* J* = 13 Hz, H-9), 5.75 (1H, d,* J* = 13 Hz, H-10), 5.55 (1H, d,* J* = 12 Hz, H-12a), 5.46 (1H, d,* J* = 12 Hz, H-12b), 4.37 (1H, s, H-6), 3.35 (3H, s, H_3_-16), 3.09 (1H, d,* J* = 10 Hz, H-2), 2.34 (1H, t,* J* = 10 Hz, H-3a), 2.11 (1H, d,* J* = 16 Hz, H-7a), 1.54 (1H, t,* J* = 10 Hz, H-3b), 1.35 (1H, d,* J* = 16 Hz, H-7b), 1.37 (3H, s, H_3_-13), 1.37 (3H, s, H_3_-14), 1.18 (3H, s, H_3_-15).

### 2.6. X-Ray Crystallographic Analysis of** 1a** [18]

Upon crystallization from MeOH/H_2_O (10 : 1) using the vapor diffusion method, colorless crystals were obtained for** 1a**, and a crystal (0.90 × 0.27 × 0.08 mm^3^) was separated from the sample and mounted on a glass fiber, and data were collected using a Rigaku Saturn CCD area detector with graphite-monochromated Mo K*α* radiation, *λ* = 0.71073 Ǻ at 173(2) K. Crystal data: C_26_H_33_F_3_O_6_·H_2_O, *M* = 516.54, space group orthorhombic,* P*2_1_2_1_2_1_; unit cell dimensions *a* = 6.9639 (14) Å, *b* = 17.211 (3) Å, *c* = 21.948 (5) Å, *V* = 2630.5 (9) Å^3^, *Z* = 4, *D*_calcd_ = 1.304 mg/m^3^,*μ* = 0.107 mm^−1^, *F*(000) = 1096. The structure was solved by direct methods using SHELXL-2016 [19] and refined using full-matrix least-squares difference Fourier techniques. All nonhydrogen atoms were refined with anisotropic displacement parameters, and all hydrogen atoms were placed in idealized positions and refined as riding atoms with the relative isotropic parameters. Absorption corrections were applied with the Siemens Area Detector Absorption Program (SADABS) [20]. The 14274 measurements yielded 4799 independent reflections after equivalent data were averaged, and Lorentz and polarization corrections were applied. The final refinement gave *R*_1_ = 0.0811 and *wR*_2_ = 0.1232 [*I* > 2*σ*(*I*)].

### 2.7. MTS Assay

The MTS assay method was the same as that we previously described [16, 21, 22].

## 3. Results and Discussion

Punctaporonin N (**1**) was obtained as a white powder with a molecular formula of C_16_H_26_O_4_ (four degrees of unsaturation), established by HRESIMS. Analysis of its NMR spectroscopic data (Table 1) revealed structural similarity to the coisolated known compound 6-hydroxypunctaporonin B (**7**) [17], except for the presence of an *O*-methyl group (*δ*_H_/*δ*_C_ 3.26/48.7) attached to C-8, rather than a free hydroxy group as found in** 7**. This observation was supported by an HMBC correlation from the *O*-methyl proton signal (H_3_-16) to the oxygenated sp^3^ quaternary carbon at 78.4 ppm (C-8). Therefore,** 1** was proposed as the C-8 methyl ether of** 7**.

**Figure 2:**
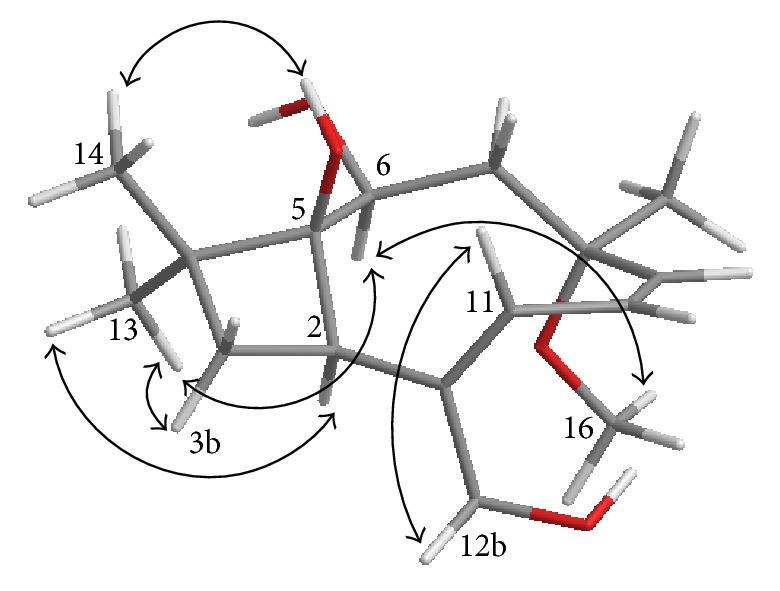
Key NOESY correlations for punctaporonin N (**1**).

The relative configuration of** 1** was determined by analysis of the ^1^H–^1^H coupling constants and NOESY data (Figure 2). The coupling constant of 13.0 Hz between H-9 and H-10, which was exactly the same as in 7, suggested the *Z*-geometry for the C-9/C-10 olefin [17]. The C-1/C-11 olefin was deduced to be *E*-configuration based on NOESY correlation of H-11 with H-12b. NOESY correlations of H_3_-13 with H-2, H-3b, and H-6 and H-6 with H_3_-16 indicated that these protons are all on the same face of the ring system, whereas that of H_3_-14 with OH-5 was used to place them on the opposite face, thereby establishing the relative configuration of** 1**.

**Figure 3:**
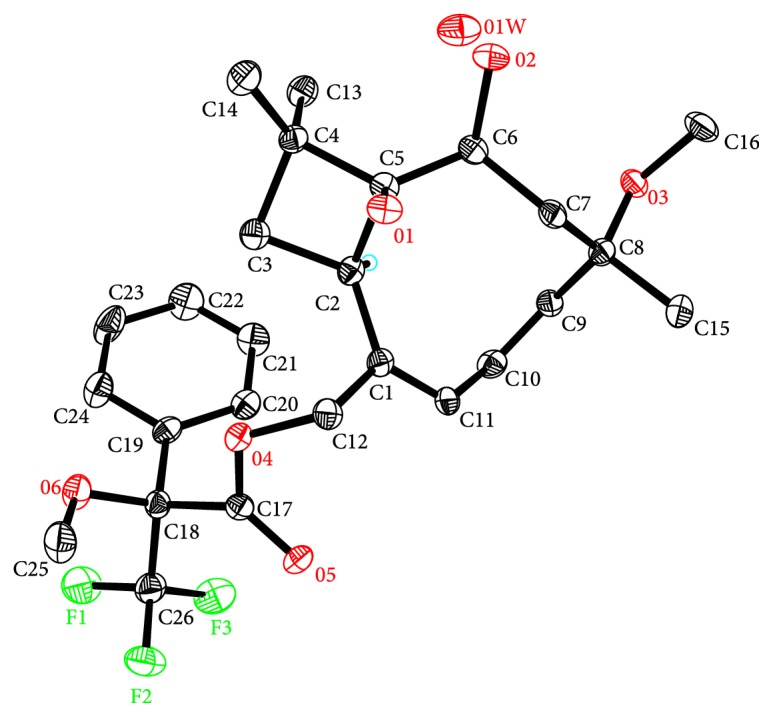
Thermal ellipsoid representation of** 1a**.

The modified Mosher method was tried to apply assigning the C-6 absolute configuration of** 1**. However, treatment of** 1** with (*R*) or (*S*)-MTPA Cl to form the C-6 MTPA ester was unsuccessful; instead the reaction products C-12 *S*-MTPA ester (**1a**) and C-12 *R*- MTPA ester (**1b**) were generated. In order to establish the absolute configuration of** 1**, one of the reaction products C-12 *S*-MTPA ester (**1a**) was purified by HPLC to obtain a single crystal, and its perspective ORTEP plot is shown in Figure 3. Although the X-ray diffraction was collected by Mo K*α* radiation, which only gave the relative configuration for** 1a**, the absolute configuration of the chiral center at C-18 generated from (*R*)-MTPA Cl had been assigned to be *S*. Therefore, the absolute configuration of** 1** was established as 2*R*, 5*S*, 6*S*, and 8*S* by single-crystal X-ray crystallographic analysis of its C-12 *S*-MTPA ester (**1a**).

Punctaporonin O (**2**) was assigned the same molecular formula C_16_H_26_O_4_ as** 1** by HRESIMS. Its NMR data (Table 2) were nearly identical to those of the coisolated known compound 6-hydroxypunctaporonin A (**8**) [17], except that the exchangeable proton at 4.18 ppm (OH-9) was replaced by a methyl group (*δ*_H_/*δ*_C_ 3.33/57.5). An HMBC correlation from the *O*-methyl proton signal (H_3_-16) to the oxymethine carbon at 97.3 ppm (C-9) suggested that** 2** is the C-9 methyl ether of** 8**. The absolute configuration of** 2** was proposed as shown by analogy to** 8**, which was also supported by the nearly identical specific rotation values recorded for both compounds [17].

The molecular formula of punctaporonin P (**3**) was determined to be C_17_H_26_O_5_ (five degrees of unsaturation) by HRESIMS, which is 28 mass units more than that of** 2**, corresponding to an extra carbonyl group. The NMR data of** 3** (Table 2) were similar to those of** 2**, except that the oxymethine proton at 3.60 ppm (H-9) was significantly downfield to 5.11 ppm in** 3**. In addition, the *O*-methyl group attached to C-9 was replaced by an acetyl unit (*δ*_H_/*δ*_C_ 2.03/21.3, 170.4), indicating that the C-9 oxygen of** 3** was acetylated. An HMBC correlation from the downfield oxymethine proton (H-9) to the carboxylic carbon at 170.4 ppm indicated that** 3** is the C-9 monoacetate of** 8**, with its configuration similarly deduced by analogy to compounds** 1** and** 2**.

Punctaporonin Q (**4**) gave a pseudomolecular ion [M + Na]^+^ peak by HRESIMS, consistent with the molecular formula C_17_H_26_O_5_ (five degrees of unsaturation), which is the same as** 3**. Its NMR spectra showed resonances similar to those of** 3**, indicating that** 4** is also a monoacetate of** 8**, but with a different position for acetylation. Specifically, the ^1^H NMR chemical shifts of H_2_-12 in** 4** (*δ*_H_ 4.37 and 4.57, Table 2) were significantly downfield compared to those in** 3** (*δ*_H_ 3.48 and 3.73), suggesting that OH-12 was acetylated. The observation was supported by an HMBC correlation from H_2_-12 to the carboxylic carbon of the acetyl unit (*δ*_C_ 171.0).

The elemental composition of punctaporonin R (**5**) was established as C_16_H_26_O_4_ (four degrees of unsaturation) by HRESIMS, which has one more CH_2_ unit than another coisolated known compound, 6-hydroxypunctaporonin E (**9**) [17]. Comparison of the NMR data of** 5** (Table 3) and** 9** readily identified** 5** as the C-11 methyl ether (*δ*_H_/*δ*_C_ 3.19/60.7) of the latter and having the same configuration as its precedent.

Punctaporonin S (**6**) was assigned the molecular formula C_19_H_28_O_6_ (six degrees of unsaturation) by HRESIMS. Its NMR spectra showed structural characteristics similar to those of** 5**, but the ^1^H NMR chemical shifts of H-11 (*δ*_H_ 5.70) and H_2_-12 (*δ*_H_ 4.06 and 4.88) were significantly downfield compared to** 5** (H-11: *δ*_H_ 3.62; H_2_-12: *δ*_H_ 3.65 and 4.06), indicating that both OH-11 and OH-12 are acetylated, which were supported by relevant HMBC correlations. Therefore,** 6** was assigned as the diacetate of the known compound** 9**, with its configuration deduced as shown.

To verify that the new metabolites** 1**–**6** are authentic natural products, a portion of the freeze-dried fermented rice substrate was extracted with distilled, HPLC grade acetone, and the resulting extract was subjected to RP HPLC analysis using distilled, HPLC grade H_2_O and MeOH as solvents. Compounds** 1–6 **were identified on the HPLC chromatogram of the crude extract by comparison of their retention times with the pure compounds, indicating that** 1–6** are indeed naturally occurring metabolites.

Compounds** 2**,** 5**, and** 6** showed modest cytotoxicity against HeLa (cervical epithelium) cells, showing IC_50_ values of 16.6, 10.4, and 47.4 *µ*M, respectively, while the positive control cisplatin showed an IC_50_ value of 7.6 *µ*M. The remaining compounds did not show noticeable cytotoxic effects against HeLa cells (IC_50_ > 100 *µ*M).

## 4. Conclusion

In summary, six new caryophyllene sesquiterpenoids, punctaporonins N–S (**1**–**6**), and three known ones, 6-hydroxypunctaporonins B (**7**), A (**8**), and E (**9**), have been isolated from solid cultures of* Cytospora *sp. The structures of** 1**–**6** were elucidated primarily by NMR spectroscopy. The absolute configuration of** 1 **was assigned by X-ray crystallographic analysis of its* S*-MTPA ester. Compounds** 2**,** 5**, and** 6** showed modest cytotoxicity against HeLa cells. These results implied that the fungi isolated from those unique and competitive niches could prove to be valuable sources of new bioactive natural products.

## Supplementary Material

NMR spectra of compounds 1–6 and X-ray crystallographic data for 1a were available in the supplementary.

## Figures and Tables

**Table 1:** NMR data (400 MHz, Acetone-*d*_6_) for punctaporonin N (**1**).

Position	*δ*_C_, mult.	*δ*_H_ (*J* in Hz)	HMBC^a^	NOESY
1	140.0, qC			
2	39.8, CH	3.39, dd (11.9, 8.2)	1, 3, 5, 11, 12	13
3a	34.0, CH_2_	2.15, dd (11.9, 9.8)	1, 2, 4, 13, 14	
3b		1.49, dd (9.8, 8.2)	2, 4, 5, 13	13
4	41.0, qC			
5	82.7, qC			
6	71.4, CH	3.86, br d (4.7)		13, 16
7a	42.5, CH_2_	2.63, dd (16.3, 4.7)	5, 6, 15	
7b		1.64, dt (16.3, 2.0)	6, 8, 9, 15	
8	78.4, qC			
9	141.9, CH	5.70, dt (13.0, 2.0)	7, 10, 11	
10	124.5, CH	5.62, d (13.0)	1, 9	
11	125.9, CH	5.88, s	1, 2, 9, 12	12b
12a	64.8, CH_2_	4.19, d (12.0)	1, 11	
12b		3.93, d (12.0)	1, 2, 11	11, 13
13	24.6, CH_3_	1.23, s	3, 4, 5, 14	2, 3b, 6, 12b
14	24.1, CH_3_	1.07, s	3, 4, 5, 13	OH-5
15	26.0, CH_3_	1.10, s	7, 8, 9	16
16	48.7, CH_3_	3.26, s	8	6, 15
OH-5		4.53, br s		14
OH-6		4.67, br s		
OH-12		3.14, br s		

^a^HMBC correlations, optimized for 8 Hz, are from proton(s) stated to the indicated carbon.

**Table 2:** NMR spectroscopic data for punctaporonins O–Q (**2**–**4**) in acetone-*d*_6_.

Position	**2**		**3**		**4**	
	*δ*_C_^a^, mult.	*δ*_H_^b^ (*J* in Hz)	*δ*_C_^a^, mult.	*δ*_H_^b^ (*J* in Hz)	*δ*_C_^a^, mult.	*δ*_H_^b^ (*J* in Hz)
1	55.3, qC		55.6, qC		53.6, qC	
2	47.1, CH	2.54, dd (13.0, 8.0)	47.2, CH	2.32, dd (12.8, 7.8)	45.6, CH	2.35, dd (12.5, 7.9)
3a	35.5, CH_2_	2.02, dd (13.0, 8.0)	35.5, CH_2_	2.08, dd (12.8, 8.9)	36.3, CH_2_	2.13, dd (12.5, 9.2)
3b		1.46, t (8.0)		1.52, dd (8.9, 7.8)		1.46, dd (9.2, 7.9)
4	42.8, qC		42.9, qC		42.7, qC	
5	80.6, qC		80.4, qC		81.7, qC	
6	68.9, CH	4.09, dd (10.0, 5.6)	68.7, CH	4.06, ddd (10.3, 7.9, 5.6)	68.9, CH	4.17, dd (10.0, 5.9)
7a	41.5, CH_2_	1.86, dd (13.5, 5.6)	41.2, CH_2_	1.77, dd (14.0, 5.6)	40.3, CH_2_	1.90, dd (13.5, 5.9)
7b		1.56, dd (13.5, 10.0)		1.62, dd (14.0, 10.3)		1.64, dd (13.5, 10.0)
8	51.0, qC		50.0, qC		50.9, qC	
9	97.3, CH	3.60, d (2.0)	89.4, CH	5.11, d (2.5)	86.5, CH	4.07, d (2.5)
10	127.9, CH	5.90, dd (6.0, 2.0)	126.6, CH	5.67, dd (5.9, 2.5)	130.6, CH	5.68, dd (6.0, 2.5)
11	143.4, CH	5.74, d (6.0)	145.3, CH	5.88, d (5.9)	141.2, CH	5.92, d (6.0)
12a	62.2, CH_2_	3.68, d (11.4)	62.0, CH_2_	3.73, d (11.2)	65.6, CH_2_	4.57, d (11.6)
12b		3.40, d (11.4)		3.48, dd (11.2, 8.0)		4.37, d (11.6)
13	23.4, CH_3_	1.11, s	23.4, CH_3_	1.19, s	23.3, CH_3_	1.10, s
14	24.1, CH_3_	1.08, s	24.0, CH_3_	1.12, s	23.7, CH_3_	1.05, s
15	24.6, CH_3_	1.05, s	24.3, CH_3_	1.10, s	26.2, CH_3_	0.84, s
16	57.5, CH_3_	3.33, s				
OH-5		4.82, br s		4.88, s		
OH-6		5.39, br s		5.37, d (7.9)		
OH-9						3.29, s
OH-12		3.25, br s		3.44, d (8.0)		
Ac-9			21.3, CH_3_	2.03, s		
			170.4, qC			
Ac-12					21.0, CH_3_	1.93, s
					171.0, qC	

^a^Recorded at 100 MHz. ^b^Recorded at 500 MHz.

**Table 3:** NMR spectroscopic data for punctaporonins R (**5**) and S (**6**) in acetone-*d*_6_.

Position	**5**		**6**	
	*δ*_C_^a^, mult.	*δ*_H_^b^ (*J* in Hz)	*δ*_C_^c^, mult.	*δ*_H_^b^ (*J* in Hz)
1	51.2, qC		53.6, qC	
2	43.8, CH	2.09, t (7.3)	39.2, CH	2.01, ddd (12.4, 7.8, 1.4)
3a	35.8, CH_2_	2.02, dd (7.3, 5.5)	37.8, CH_2_	2.37, dd (12.4, 9.2)
3b		1.54, t (5.5)		1.73, dd (9.2, 7.8)
4	42.1, qC		42.2, qC	
5	80.2, qC		82.6, qC	
6	69.3, CH	3.47, dd (11.3, 7.3)	69.0, CH	3.66, dd (10.2, 5.5)
7a	42.9, CH_2_	1.71, d (11.3)	42.4, CH_2_	1.94, dd (12.2, 5.5)
7b				1.86, dd (12.2, 10.2)
8	53.6, qC		48.8, qC	
9	149.2, CH	5.85, d (5.8)	150.0, CH	6.01, d (5.7)
10	126.8, CH	5.82, dd (5.8, 2.2)	126.8, CH	5.70, dd (5.7, 2.7)
11	90.9, CH	3.62, d (2.2)	81.3, CH	5.28, d (2.7)
12a	57.3, CH_2_	3.65, d (11.4)	63.6, CH_2_	4.88 d (10.2)
12b		4.06, dd (11.4, 7.0)		4.06, dd (10.2, 1.4)
13	23.3, CH_3_	1.10, s	22.9, CH_3_	1.14, s
14	24.2, CH_3_	1.08, s	23.6, CH_3_	1.10, s
15	27.3, CH_3_	1.18, s	27.9, CH_3_	1.11, s
16	60.7, CH_3_	3.19, s		
OH-5		5.29, br s		
OH-6		5.18, d (7.3)		3.69, (5.5)
OH-12		3.09, d (7.0)		
Ac-11			20.8, CH_3_	1.93, s
			170.1, qC	
Ac-12			21.0, CH_3_	1.95, s
			170.9, qC	

^a^Recorded at 150 MHz. ^b^Recorded at 500 MHz. ^c^Recorded at 100 MHz.
