# Supplementary material for: Punctaporonins N–S, New Caryophyllene Sesquiterpenoids from Cytospora sp
Source: Biomed Res Int. 2017 Oct 24;2017:7871459. doi: 10.1155/2017/7871459 (PMC5674490; doi:10.1155/2017/7871459)
Supplement: Supplementary file 1 — NMR spectra of compounds 1–6 and X-ray crystallographic data for 1a were available in the supplementary. [file 7871459.f1.zip › new supplementary materials/Supporting Information.doc]

**Punctaporonins N–S, New Caryophyllene Sesquiterpenoids from *Cytospora* sp.**

Yan Li,^†,‡^ Qingbin Wang,^†^ Xingzhong Liu,^&^ and Yongsheng Che*^,†^

^†^State Key Laboratory of Toxicology & Medical Countermeasures, Beijing Institute of Pharmacology & Toxicology, Beijing 100850, China

^‡^Institute of Vegetables and Flowers, Chinese Academy of Agricultural Sciences, Beijing 100081, China

^&^State Key Laboratory of Mycology, Institute of Microbiology, Chinese Academy of Sciences, Beijing 100190, China

**Contents**	**Page**

1) **Figure S1.** ^1^H NMR spectrum of punctaporonin N (**1**; 500 MHz, acetone-*d*_6_)	2

2) **Figure S2.** ^13^C NMR spectrum of punctaporonin N (**1**; 100 MHz, acetone-*d*_6_)	3

3) **Figure S3.** ^1^H NMR spectrum of punctaporonin O (**2**; 500 MHz, acetone-*d*_6_)	4

4) **Figure S4.** ^13^C NMR spectrum of punctaporonin O (**2**; 100 MHz, acetone-*d*_6_)	5

5) **Figure S5.** ^1^H NMR spectrum of punctaporonin P (**3**; 500 MHz, acetone-*d*_6_)	6

6) **Figure S6.** ^13^C NMR spectrum of punctaporonin P (**3**; 100 MHz, acetone-*d*_6_)	7

7) **Figure S7.** ^1^H NMR spectrum of punctaporonin Q (**4**; 500 MHz, acetone-*d*_6_)	8

8) **Figure S8.** ^13^C NMR spectrum of punctaporonin Q (**4**; 100 MHz, acetone-*d*_6_)	9

9) **Figure S9.** ^1^H NMR spectrum of punctaporonin R (**5**; 500 MHz, acetone-*d*_6_)	10

10) **Figure S10.** ^13^C NMR spectrum of punctaporonin R (**5**; 150 MHz, acetone-*d*_6_)	11

11) **Figure S11.** ^1^H NMR spectrum of punctaporonin S (**6**; 500 MHz, acetone-*d*_6_)	12

12) **Figure S12.** ^13^C NMR spectrum of punctaporonin S (**6**; 100 MHz, acetone-*d*_6_)	13

13) **Table S1.** NMR Spectroscopic Data (Acetone-*d*_6_) for **2**	14

14) **Table S2.** NMR Spectroscopic Data (Acetone-*d*_6_) for **3**	15

15) **Table S3.** NMR Spectroscopic Data (Acetone-*d*_6_) for **4**	16

16) **Table S4.** NMR Spectroscopic Data (Acetone-*d*_6_) for **6**	17

**Figure S1.** ^1^H NMR Spectrum of Punctaporonin N (**1**; 500 MHz, Acetone-*d*_6_)

**Figure S2.** ^13^C NMR Spectrum of Punctaporonin N (**1**; 100 MHz, Acetone-*d*_6_)

**Figure S3.** ^1^H NMR Spectrum of Punctaporonin O (**2**; 500 MHz, Acetone-*d*_6_)

**Figure S4.** ^13^C NMR Spectrum of Punctaporonin O (**2**; 100 MHz, Acetone-*d*_6_)

**Figure S5.** ^1^H NMR Spectrum of Punctaporonin P (**3**; 500 MHz, Acetone-*d*_6_)

**Figure S6.** ^13^C NMR Spectrum of Punctaporonin P (**3**; 100 MHz, Acetone-*d*_6_)

**Figure S7.** ^1^H NMR Spectrum of Punctaporonin Q (**4**; 500 MHz, Acetone-*d*_6_)

**Figure S8.** ^13^C NMR Spectrum of Punctaporonin Q (**4**; 100 MHz, Acetone-*d*_6_)

**Figure S9.** ^1^H NMR Spectrum of Punctaporonin R (**5**; 500 MHz, Acetone-*d*_6_)

**Figure S10.** ^13^C NMR Spectrum of Punctaporonin R (**5**; 150 MHz, Acetone-*d*_6_)

**Figure S11.** ^1^H NMR Spectrum of Punctaporonin S (**6**; 500 MHz, Acetone-*d*_6_)

**Figure S12.** ^13^C NMR Spectrum of Punctaporonin S (**6**; 100 MHz, Acetone-*d*_6_)

**Table S1.** NMR Spectroscopic Data (Acetone-*d*_6_) for **2**.

| position | *δ*_C_, mult. | *δ*_H_ (*J* in Hz) | HMBC*^a^* | NOESY |
| --- | --- | --- | --- | --- |
| 1 | 55.3, qC |  |  |  |
| 2 | 47.1, CH | 2.54, dd (13.0, 8.0) | 1, 3, 4, 5, 6, 11, 12 | 6, 13 |
| 3a | 35.5, CH_2_ | 2.02, dd (13.0, 8.0) | 2, 4, 13, 14 | 14 |
| 3b |  | 1.46, t (8.0) | 2, 4, 5, 13 |  |
| 4 | 42.8, qC |  |  |  |
| 5 | 80.6, qC |  |  |  |
| 6 | 68.9, CH | 4.09, dd (10.0, 5.6) | 7 | 2, 13 |
| 7a | 41.5, CH_2_ | 1.86, dd (13.5, 5.6) | 1, 5, 6, 8, 9 |  |
| 7b |  | 1.56, dd (13.5, 10.0) | 6, 8, 9, 15 |  |
| 8 | 51.0, qC |  |  |  |
| 9 | 97.3, CH | 3.60, d (2.0) | 1, 11, 16 | 15 |
| 10 | 127.9, CH | 5.90, dd (6.0, 2.0) | 1, 8, 9, 11 |  |
| 11 | 143.4, CH | 5.74, d (6.0) | 1, 8, 9, 10, 12 | 12a |
| 12a | 62.2, CH_2_ | 3.68, d (11.4) | 1, 2, 8 | 11 |
| 12b |  | 3.40, d (11.4) |  | 15 |
| 13 | 23.4, CH_3_ | 1.11, s | 3, 4, 5, 14 | 2, 6 |
| 14 | 24.1, CH_3_ | 1.08, s | 3, 4, 5, 13 | 3a |
| 15 | 24.6, CH_3_ | 1.05, s | 1, 7, 8, 9 | 9, 12b |
| 16 | 57.5, CH_3_ | 3.33, s | 9 |  |
| OH-5 |  | 4.82, br s |  | OH-6, OH-12 |
| OH-6 |  | 5.39, br s |  | OH-5,OH-12 |
| OH-12 |  | 3.25, br s |  | OH-5, OH-6 |

*^a^*HMBC correlations, optimized for 8 Hz, are from proton(s) stated to the indicated carbon.

**Table S2.** NMR Spectroscopic Data (Acetone-*d*_6_) for **3**

| position | *δ*_C_, mult. | *δ*_H_ (*J* in Hz) | HMBC*^a^* |  | NOESY |
| --- | --- | --- | --- | --- | --- |
| 1 | 55.6, qC |  |  |  |  |
| 2 | 47.2, CH | 2.32, dd (12.8, 7.8) | 1, 3, 5, 6, 12 |  | 13 |
| 3a | 35.5, CH_2_ | 2.08, dd (12.8, 8.9) | 2, 4, 13 |  | 14 |
| 3b |  | 1.52, dd (8.9, 7.8) | 2, 4, 5, 13 |  | 13 |
| 4 | 42.9, qC |  |  |  |  |
| 5 | 80.4, qC |  |  |  |  |
| 6 | 68.7, CH | 4.06, ddd (10.3, 7.9, 5.6) |  |  |  |
| 7a | 41.2, CH_2_ | 1.77, dd (14.0, 5.6) | 1, 5, 6, 8, 9 |  |  |
| 7b |  | 1.62, dd (14.0, 10.3) | 6, 8, 9, 15 |  | 15 |
| 8 | 50.0, qC |  |  |  |  |
| 9 | 89.4, CH | 5.11, d (2.5) | 1, 11, 15, 17 |  | 15 |
| 10 | 126.6, CH | 5.67, dd (5.9, 2.5) | 1, 8, 9 |  |  |
| 11 | 145.3, CH | 5.88, d (5.9) | 1, 8, 9, 10 |  |  |
| 12a | 62.0, CH_2_ | 3.73, d (11.2) | 1, 2, 8, 11 |  |  |
| 12b |  | 3.48, dd (11.2, 8.0) | 1, 2, 11 |  | 15 |
| 13 | 23.4, CH_3_ | 1.19, s | 3, 4, 5, 14 |  | 2, 3b, 14 |
| 14 | 24.0, CH_3_ | 1.12, s | 3, 4, 5, 13 |  | 3a, 13 |
| 15 | 24.3, CH_3_ | 1.10, s | 1, 7, 8, 9 |  | 7b, 9,12b, OH-12 |
| 16 | 21.3, CH_3_ | 2.03, s | 17 |  |  |
| 17 | 170.4, qC |  |  |  |  |
| OH-5 |  | 4.88, s |  |  | OH-6, OH-12 |
| OH-6 |  | 5.37, d (7.9) |  |  | OH-5, OH-12 |
| OH-12 |  | 3.44, d (8.0) |  |  | OH-5, OH-6, 15 |

*^a^*HMBC correlations, optimized for 8 Hz, are from proton(s) stated to the indicated carbon.

**Table S3.** NMR Spectroscopic Data (Acetone-*d*_6_) for **4**

| position | *δ*_C_, mult. | *δ*_H_ (*J* in Hz) | HMBC*^a^* | NOESY |
| --- | --- | --- | --- | --- |
| 1 | 53.6, qC |  |  |  |
| 2 | 45.6, CH | 2.35, dd (12.5, 7.9) | 1, 3, 5, 6, 12 | 6, 13 |
| 3a | 36.3, CH_2_ | 2.13, dd (12.5, 9.2) | 2, 4, 13 | 12a, 14 |
| 3b |  | 1.46, dd (9.2, 7.9) | 2, 4, 5, 13 | 13 |
| 4 | 42.7, qC |  |  |  |
| 5 | 81.7, qC |  |  |  |
| 6 | 68.9, CH | 4.17, dd (10.0, 5.9) |  | 2, 13 |
| 7a | 40.3, CH_2_ | 1.90, dd (13.5, 5.9) | 1, 5, 6, 8, 9 | 6, 15 |
| 7b |  | 1.64, dd (13.5, 10.0) | 6, 8, 9, 15 | 15 |
| 8 | 50.9, qC |  |  |  |
| 9 | 86.5, CH | 4.07, d (2.5) | 1, 11, 15 | 15 |
| 10 | 130.6, CH | 5.68, dd (6.0, 2.5) | 1, 8, 9 |  |
| 11 | 141.2, CH | 5.92, d (6.0) | 1, 8, 9, 10 |  |
| 12a | 65.6, CH_2_ | 4.57, d (11.6) | 1, 2, 8, 11, 17 | 3a |
| 12b |  | 4.37, d (11.6) | 1, 2, 11, 17 | 15 |
| 13 | 23.3, CH_3_ | 1.10, s | 3, 4, 5, 14 | 2, 3b, 6 |
| 14 | 23.7, CH_3_ | 1.05, s | 3, 4, 5, 13 | 3a |
| 15 | 26.2, CH_3_ | 0.84, s | 1, 7, 8, 9 | 7a, 7b, 9, 12b |
| 16 | 21.0, CH_3_ | 1.93, s | 17 |  |
| 17 | 171.0, qC |  |  |  |
| OH-9 |  | 3.29, s |  |  |

*^a^*HMBC correlations, optimized for 8 Hz, are from proton(s) stated to the indicated carbon.

**Table S4.** NMR Spectroscopic Data (Acetone-*d*_6_) for **6**

| position | *δ*_C_, mult. | *δ*_H_ (*J* in Hz) | HMBC*^a^* | NOESY |
| --- | --- | --- | --- | --- |
| 1 | 53.6, qC |  |  |  |
| 2 | 39.2, CH | 2.01, ddd (12.4, 7.8, 1.4) | 3, 4, 6, 8, 11, 12 | 6, 11, 13 |
| 3a | 37.8, CH_2_ | 2.37, dd (12.4, 9.2) | 2, 4, 13 | 14 |
| 3b |  | 1.73, dd (9.2, 7.8) | 2, 4, 14 | 11, 13 |
| 4 | 42.2, qC |  |  |  |
| 5 | 82.6, qC |  |  |  |
| 6 | 69.0, CH | 3.66, dd (10.2, 5.5) |  | 2, 13 |
| 7a | 42.4, CH_2_ | 1.94, dd (12.2, 10.2) | 1, 5, 6, 8, 9, 15 |  |
| 7b |  | 1.86, dd (12.2, 5.5) | 1, 5, 6, 8, 9 | 9 |
| 8 | 48.8, qC |  |  |  |
| 9 | 150.0, CH | 6.01, d (5.7) | 1, 7, 8, 10, 11 | 7b, 15 |
| 10 | 126.8, CH | 5.70, dd (5.7, 2.7) | 1, 8, 9, 11 |  |
| 11 | 81.3, CH | 5.28, d (2.7) | 1, 2, 9, 19 | 2, 3b |
| 12a | 63.6, CH_2_ | 4.88 d (10.2) | 2, 8, 11, 17 | 15 |
| 12b |  | 4.06, dd (10.2, 1.4) | 2, 8, 11, 17 | 15 |
| 13 | 22.9, CH_3_ | 1.14, s | 3, 4, 5, 14 | 2, 3b, 6 |
| 14 | 23.6, CH_3_ | 1.10, s | 3, 4, 5, 13 | 3a, 12a, 12b |
| 15 | 27.9, CH_3_ | 1.11, s | 1, 7, 8, 9 | 9 |
| 16 | 21.0, CH_3_ | 1.95, s | 17 |  |
| 17 | 170.9, qC |  |  |  |
| 18 | 20.8, CH_3_ | 1.93, s | 19 |  |
| 19 | 170.1, qC |  |  |  |
| OH-6 |  | 3.69, (5.5) |  |  |

*^a^*HMBC correlations, optimized for 8 Hz, are from proton(s) stated to the indicated carbon.
